# The Pillars for Renal Disease Treatment in Patients with Type 2 Diabetes

**DOI:** 10.3390/pharmaceutics15051343

**Published:** 2023-04-27

**Authors:** Jessica Kearney, Luigi Gnudi

**Affiliations:** 1 Department of Diabetes and Endocrinology, Guy’s and St Thomas NHS Foundation Trust, London SE1 9RT, UK; 2 School of Cardiovascular and Metabolic Medicine & Sciences, British Heart Foundation Centre of Research Excellence, Faculty of Life Sciences & Medicine, King’s College London, London WC2R 2LS, UK

**Keywords:** diabetes, kidney disease, therapeutics

## Abstract

The diabetes epidemic and the increasing number of patients with diabetic chronic vascular complications poses a significant challenge to health care providers. Diabetic kidney disease is a serious diabetes-mediated chronic vascular complication and represents a significant burden for both patients and society in general. Diabetic kidney disease not only represents the major cause of end stage renal disease but is also paralleled by an increase in cardiovascular morbidity and mortality. Any interventions to delay the development and progression of diabetic kidney disease are important to reduce the associated cardiovascular burden. In this review we will discuss five therapeutic tools for the prevention and treatment of diabetic kidney disease: drugs inhibiting the renin–angiotensin–aldosterone system, statins, the more recently recognized sodium-glucose co-transporter-2 inhibitors, glucagon-like peptide 1 agonists, and a novel non-steroidal selective mineralocorticoid receptor antagonist.

## 1. Introduction

Diabetic kidney disease (DKD) is the main cause of end-stage renal disease (ESRD) and, alongside drugs inhibiting the renin–angiotensin–aldosterone (RAAS) system and statins, novel pharmacological agents have recently been proposed to target renal disease in diabetes.

Sodium-glucose co-transporter-2 (SGLT2) inhibitors, glucagon-like peptide 1 (GLP1) receptor agonists, and non-steroidal mineralocorticoid receptor antagonists have recently been shown to have important renoprotective effects in patients with diabetes, which is also paralleled by a significant and important reduction in cardiovascular morbidity and mortality [1,2,3].

We discuss the five therapeutic tools or ‘pillars’ that can be used for DKD in addition to how recent advances could contribute to the discovery of novel mechanisms of action and the development of new treatment options in the future.

## 2. Diabetic Kidney Disease

Global diabetes prevalence is expected to rise to 12.2% (783.2 million), and its related health cost has been projected to reach USD 1054 billion by 2040 [4]. Of the diabetic population, approximately one-third will develop DKD, an estimate similar in both type 1 (T1DM) and type 2 diabetes (T2DM). Patients with T2DM represent an older patient group with more frequent co-existence of pathologies such as cardiovascular disease [5].

Diabetes represents the most common cause of ESRD worldwide [5]. Declining renal function and albuminuria are independently and additively associated with an increase in cardiovascular morbidity and mortality [6,7,8] which is 2–3 times higher than that seen in patients with diabetes but without DKD [9].

The clinical presentation of DKD is typically characterized by glomerular hyperfiltration, followed by albuminuria, overt proteinuria, hypertension, and progressive renal function decline that leads to ESRD [10,11]. Most patients with diabetes present with classical features of DKD as described, but at times renal function decline is not paralleled by albuminuria, an event mainly observed in T2DM [12,13,14].

Poor glycemic control drives the development and progression of DKD, and studies in T1DM have demonstrated that good metabolic control prevents and delays the progression of DKD [15]. Good metabolic control prevents hyperfiltration, which is believed to be a risk factor for CKD progression [16,17,18,19]. Glomerular hyperfiltration reflects an increase in glomerular capillary pressure that translates in anatomo-structural damage. Even in normotensive conditions, glomerular capillary hypertension synergizes with metabolic perturbations and drives the development and progression of DKD [20,21,22].

Mechanisms of hyperfiltration have been related to two main mechanisms: an upregulation of SGLT2 with increased glucose and sodium reabsorption at the level of the proximal tubule and increased glomerular expression/secretion of angiotensin-2 [23,24].

Increased sodium and glucose reabsorption at the level of the proximal tubule results in a reduced amount of sodium to the macula densa that, by tubule-glomerular feedback, leads to glomerular afferent arteriolae vasodilation [25,26,27,28]. In addition, the local activation of the RAAS with increased levels of angiotensin-2 leads to an increase in efferent arteriolae vasoconstriction and secondary glomerular hypertension [22,29,30].

It is recognized that the risk of development and progression of DKD lies mainly on poor glycemic and blood pressure control and their interaction synergize in driving renal damage [22]. Obesity, mainly visceral, also represents an important factor for DKD progression [31]. Obesity results in RAAS activation and hyperfiltration [32,33], while weight loss improves altered glomerular hemodynamics in diabetes [34,35]. Dyslipidemia has also been implicated in the pathophysiology of DKD and for the development of albuminuria, with statins proving to be an effective treatment [36,37,38]. Similarly, for fibrates there seem to be a positive effect on renal function decline and albuminuria [39,40].

## 3. Therapeutic Strategies for DKD

The key approach to reduce DKD-mediated ESRD is to prevent and delay the renal function decline, as once a fall in renal function occurs, it is difficult to regain, apart from when normoglycemic conditions are implemented for a long time, such as following pancreas transplantation [41].

### 3.1. Lifestyle

Lifestyle measures are a key component in the overall management of DKD. Dietary advice is important in the management of both CKD and diabetes. In CKD, the National Kidney Foundation guidelines suggest a registered dietician nutritionist’s intervention to provide nutritional advice [42]. Considerations include reducing dietary protein intake, ensuring adequate fruit and vegetables, following a Mediterranean diet, and considering the need for vitamin and mineral supplements [42]. Depending on the patient, advice to reduce potassium intake and restrict oral fluids may be given [42].

There is no specific recommended diet for diabetes, but general advice includes eating a variety of fruit and vegetables, reducing carbohydrates, saturated fats, and salts, and choosing low- over high-glycemic-index foods [43]. A very-low-calorie diet (825–853 kcal/day) has been shown to lead to remission of T2DM in almost half of patients at 12 months [44]. A very low carbohydrate diet has been shown to improve glycemic control but not renal outcomes in DKD [45]. In addition to diet, regular physical activity, not smoking and weight loss for patients with obesity are advisable in DKD [46].

**Figure 1:**
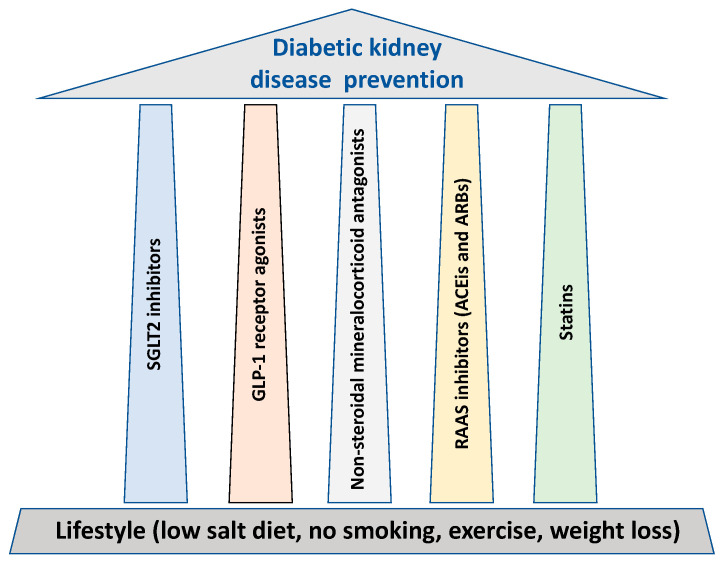
The treatment “pillars” for renal protection in diabetes.

In parallel to lifestyle, glycemic and blood pressure control, all cornerstones for the prevention of DKD, we outline five major treatment “pillars” that possess major renal protective properties (Figure 1).

### 3.2. SGLT2 Inhibitors

SGLT2 inhibitors act to reduce proximal tubular glucose reabsorption from the renal glomerular filtrate [47]. SGLT2 is an energy-dependent sodium-coupled glucose transporter expressed mainly in the S1 and S2 segment of the nephron proximal tubule [48,49]. SGLT2 is upregulated in diabetes and contributes to both proximal tubule glucose and sodium reabsorption [26]. The SGLT2 inhibitors were initially developed as oral hypoglycemic agents. By reducing glucose proximal tubule reabsorption, SGLT2 inhibitors lead to an increase in urinary glucose excretion, promote weight loss of approximately 4–5 Kg, lower plasma glucose concentrations, and lead to a reduction in HbA1c of around 1.0% [50].

SGLT2 inhibitors were found to confer both cardiovascular and renal protection; clinical trials have demonstrated that SGLT2 inhibitor treatment results in a 30–40% relative risk reduction of cardiovascular death and hospitalization, which was mainly driven by reduction of heart failure [51,52,53]. The EMPAREG trial was the first to suggest a renoprotective role for the SGLT2 inhibitor empagliflozin [54]. Later, other studies such as the CANVAS and CANVAS-R showed a promising renoprotective effect of the SGLT2 antagonist canagliflozin [55]. Subsequently, a prospective study, the CREDENCE trial, enrolled patients with type 2 diabetes and albuminuria (albumin/creatinine ration 300–5000 mg/g) and chronic kidney disease (glomerular filtration rate: GFR of 30–90 mL/min/1.73 m^2^) that were randomized to the SGLT2 inhibitor canagliflozin at a dose of 100 mg daily or placebo. All patients were treated with RAAS inhibitors. The trial was stopped earlier due to clear benefit of patients enrolled in the treatment arm. Patient on SGLT2 inhibitor had a relative 30% reduction of a renal endpoint defined as ESRD, doubling of the serum creatinine level, or death from renal or cardiovascular causes when compared to the placebo arm [56]. Interestingly, other studies have confirmed these results in patients with T2DM and have demonstrated that the SGLT2-inhibitor-mediated renoprotective effects occurs also in the non-diabetic population [57].

The latest NICE guidelines recommend the use of SGLT2 inhibitors with metformin as dual therapy first line for patients with diabetes with established cardiovascular disease, and advise to consider their use in patients with high risk of cardiovascular disease [58]. In addition to documented weight reduction, the cardiovascular protective effects of SGLT2 inhibitors include reduction in blood pressure [59] and reduced risk of both new heart failure and of worsening existing heart failure in patients with reduced or preserved ejection fraction [51,52,53,60,61,62].

SGLT2 inhibitors, including empagliflozin, dapagliflozin, canagliflozin, and ertugliflozin, have been observed to have beneficial renal effects in several cardiovascular outcome trials. Meta-analyses of these data have demonstrated that in diabetic patients, SGLT2 inhibitors reduce the risk of progression of renal disease, ESRD, or death from renal causes [3].

The 2022 KDIGO guidelines for diabetes management in CKD recommend the use of metformin and SGLT2 inhibitors for all patients with T2DM, CKD, and an GFR > 30 mL/min/1.73 m^2^ [63]. The glucose lowering effects are attenuated at GFR < 60 mL/min/1.73 m^2^ and minimal at GFR < 30 mL/min/1.73 m^2^ [64]. However, the renal and cardiovascular benefits are seen at any GFR, and guidelines advise continuing SGLT2 inhibitors even if GFR falls below 20 mL/min/1.73 m^2^, until renal replacement therapy is initiated [56]. The cardio-renal protective effects appear within weeks/months and seem to be independent of the improvement in glycemic control. The SGLT2 beneficial effects are likely to be driven by a hemodynamic mechanism; the main proposed mechanisms linked with renal benefits include: increased diuresis and improvement in blood pressure, tubulo-glomerular feedback leading to reduced intraglomerular pressure, increased tubular oxygenation, and reduced inflammation and fibrosis [65].

#### Side Effects

An initial drop in eGFR (driven by hemodynamic changes in the glomerular circulation) is seen when SGLT2 inhibitors are initiated; however, there has been no confirmed increased risk of acute kidney injury (AKI) occurrence, and conversely, they have been seen to reduce the risk of AKI in patients with T2DM [66]. Due to the increased glucosuria, SGLT2 inhibitors increase the risk of genital infections and so patients should be educated with regards to the importance of hydration and personal hygiene [67]. An increased risk of fractures and amputations has been documented, although evidence is mixed, and further evaluation is needed to confirm these associations [67]. Similarly, an association has been made with SGLT2 inhibitors and Fournier’s gangrene, but no causality has been established [67].

SGLT2 inhibitors lead to increased lipolysis and glucagonemia, and therefore, it has been recommended that they are held peri-operatively and at times of dehydration and that ketones be monitored due to the risk of ketosis in the peri-operative setting [68,69]. Healthcare professionals are advised to educate patients on the ‘sick-day rules’ when initiating SGLT2 inhibitors to enable prevention and recognition of potential ketoacidosis. If patients are unwell or unable to eat and drink as normal, they should omit the SGLT2 inhibitor and keep themselves as well hydrated as possible; patients should be informed of the symptoms of diabetic ketoacidosis and to attend Accident & Emergency department should they develop them [70]. SGLT2 inhibitors should not be restarted until eating normally for at least 24 h and never restarted if a patient develops ketoacidosis while taking them [70].

### 3.3. GLP1 Receptor Agonists

GLP1 is an incretin hormone produced in the distal small bowel and colon that triggers the release of insulin in response to oral glucose intake. Incretin hormones slow gastric emptying and increase natriuresis and diuresis [71]. Native GLP1 has a short half-life as it is cleaved by dipeptidyl-peptidase IV (DPP IV) enzymes and eliminated renally. The GLP1 receptor agonists are synthetized from either exendin-4 from the saliva of the Gila monster, a species of venomous lizard, or human GLP1 analogues, both of which are resistant to the DPP IV degradation. The exendin-4 based agents, such as exenatide and lixisenatide, have a short half-life but strongly inhibit gastric emptying and cause a greater reduction in post-prandial hyperglycemia [72].

GLP1 receptor agonists are a group of drugs used to treat T2DM and lead to a reduction in HbA1c. GLP1 receptors are present in many tissues, but with regards to the therapeutic effects on diabetes, their activation is thought to increase insulin secretion, and reduce beta cell apoptosis and glucagon release in a glucose-dependent way [71]. Furthermore, GLP1 receptor agonists bind the GLP1 receptor present in the central nervous system and the gastrointestinal tract and favor weight loss in obese patients through an increase in satiety, reducing appetite, delaying gastric emptying, and potentially increasing thermogenesis of brown adipose tissue [73,74].

Data from clinical trials suggest that GLP1 receptor agonists improve surrogate renal endpoints, plausibly beyond the effects of improved glycemic control [75]. Exendin-4-based GLP1 receptor agonists are renally excreted and so their use is contraindicated at an eGFR < 30 mL/min/1.73 m^2^ [76]. Meanwhile, the human GLP1 receptor agonists dulaglutide, semaglutide, and liraglutide are longer-acting and are more effective at reducing fasting blood sugar and HbA1C [77]. They are not eliminated by the kidneys and so can be safely used down to an eGFR of 15 mL/min/1.73 m^2^ [76].

Cardiovascular outcome trials have shown that GLP1 receptor agonists are efficacious in reducing the risk of cardiovascular events (stroke, myocardial infarction, or cardiovascular death) in patients with T2DM [1]. The same trials have shown that GLP1 receptor agonists prevent worsening of renal function in patients with T2DM. This was measured as a composite outcome of ≥40% decline in eGFR or doubling of creatinine, need for renal replacement therapy, new macroalbuminuria, or death from renal disease [1]. The FLOW trial will be the first dedicated kidney outcome trial to evaluate whether the GLP1 receptor agonist semaglutide delays progression of kidney disease and reduces the risk of death from renal or cardiovascular disease in patients with T2DM and CKD (ClinicalTrials.gov Identifier: NCT03819153). Data are lacking on patients with ESRD on hemodialysis or renal transplant patients.

Clinical trials on DPP IV inhibitors, which prevent GLP1 degradation, have not shown a cardiovascular benefit for patients with T2DM [78]. The reasons behind this observation are unknown but could relate to the "physiological" concentration of circulating GLP1 achieved in patients treated with DPP IV inhibitors versus the "supraphysiological" levels observed with GLP-1 receptor agonists [79].

Not only do GLP1 receptor agonists improve glycemic control and stimulate weight loss, but evidence suggests they also confer nephroprotection via interaction with the renal cells, with GLP1 receptors thought to be present in the kidney [76]. The proposed mechanisms of renal protection include a reduction in oxidative stress, fibrosis, inflammation, and possibly increased natriuresis [80]. Guidelines (KDIGO) advise the use of GLP1 receptor agonists in patients with T2DM and CKD who have not achieved glycemic targets with metformin and SGLT2 inhibitors [63].

#### Side Effects

GLP1 receptor agonists do not cause hypoglycemia; however, if a patient is already on insulin or a sulphonylurea, the doses of these may need to be reduced to avoid hypoglycemia [81]. The most common side effects of GLP1 receptor agonists are gastrointestinal such as nausea, vomiting, reflux, diarrhea, or constipation [81,82]. Proposed methods of managing these include educating patients, escalating dosing more slowly if indicated, increasing hydration, reducing portion size, and considering switching to an alternative GLP1 receptor agonist [82]. Associations have been made between GLP1 receptor agonists and pancreatitis, pancreatic cancer or thyroid cancer; however, current evidence does not support a causative link [73].

### 3.4. Non-Steroidal Mineralocorticoid Receptor Antagonists

Activation of mineralocorticoid receptors leads to inflammation and fibrosis contributing to progression of CKD and cardiovascular dysfunction [69]. While recommended, the use of steroidal mineralocorticoid receptor antagonists (MRA) for patients with CKD and heart failure is often limited due to concerns of hyperkalemia, gynecomastia, impotence, and menstrual disturbances. Finerenone is a non-steroidal, selective MRA with low affinity for androgen, glucocorticoid, progesterone, and estrogen receptors and high selectivity for mineralocorticoid receptors [83,84]. It said to be more effective at reducing the pathological processes contributing to CKD than steroidal MRAs, with theoretically lower side effects [85].

Finerenone binds similarly to its receptor both in the heart and kidney when compared to the steroidal MRA spironolactone, which is preferentially distributed in the kidneys. Compared with eplerenone, another steroidal MRA, finerenone holds more potent anti-inflammatory and anti-fibrotic effects on the heart and kidney in experimental animal models. Non-steroidal MRAs have a better benefit–risk ratio than steroidal MRAs, with a reduced risk for hyperkalemia [85].

In two major phase 3 trials, FIDELIO-DKD and FIGARO-DKD, conducted in patients with T2DM and chronic kidney disease, finerenone treatment on top of maximally tolerated RAAS inhibitor treatment was shown to confer both renoprotection (composite of >40% reduction in eGFR, kidney failure, or death from renal cause) and cardioprotection (composite of cardiovascular death, non-fatal myocardial infarction or stroke, hospitalization for heart failure) [86,87]. In FIDELIO-DKD, patients either had a urine albumin–creatinine ratio (UACR) of 30–300 mg/g with an GFR of 25–60 mL/min/1.73 m^2^ of body surface area as well as diabetic retinopathy or a UACR of 300–5000 mg/g with an GFR of at least 25–75 mL/min/1.73 m^2^ [86]. The inclusion criteria of FIGARO-DKD were similar, with patients either having a UACR of 30–300 mg/g with an GFR of 25–90 mL/min/1.73 m^2^ or a UACR of 300–5000 mg/g with an GFR of at least 60 mL/min/1.73 m^2^ [87]. Patients with symptomatic heart failure with reduced ejection fraction were not eligible for inclusion in these trials.

The results of these two trials were combined in the pre-specified FIDELITY analysis, which confirmed the clinical cardiac [hazard ratio (HR): 0.86, 95% confidence interval (CI): 0.78, 0.95] and renal (HR: 0.77, 95% CI: 0.67, 0.88) protective properties of finerenone [88]. Cardio- and renaoprotective effects seemed independent of change in blood pressure as finerenone conferred only a modest effect on blood pressure [86,87,88].

#### Side Effects

A meta-analysis of the use of finerenone (when used in combination with another RAAS blockade agent) demonstrated an increased incidence of hyperkalemia compared with placebo but lower than with spironolactone. The increase in potassium plasma levels observed was moderate at only 0.17 mmol/L; however, potassium monitoring is advised [83]. NICE guidelines state finerenone should not be initiated at an eGFR of <25 mL/min/1.73 m^2^ and it should be discontinued if GFR < 25 mL/min/1.73 m^2^ [76]. Other listed side-effects are hypotension and pruritus [89].

### 3.5. RAAS Inhibitors

The RAAS is central to blood pressure regulation and fluid and electrolyte balance. RAAS inhibition with angiotensin-converting enzyme inhibitors (ACEis) and angiotensin-2 receptor blockers (ARBs) reduces the activity of angiotensin and thereby leads to vasodilation, reduced sympathetic adrenergic activity, increased natriuresis and diuresis, and inhibition of cardiac and vascular remodeling. Blood pressure control is effective against the progression of DKD [37] and ACEis and ARBs are the first-line treatment for patients with DKD [37]. RAAS blockade has been shown reduce albuminuria and to be renoprotective for this patient group [90]. The benefits to renal function that these medications provide exceed that attributable to blood pressure lowering [90]. However, double RAAS blockade with ACEis/ARBs has been associated with an increased risk of acute kidney injury and hyperkalemia [91]. In patients with T2DM on ACEIs, ARBs, or combination treatment, the addition of a mineralocorticoid antagonist results in ~50% reduction in albuminuria [92] and retains the renoprotective effect [86,93,94].

#### Side Effects

Adverse effects of ACEis and ARBs include hyperkalemia, renal impairment, angio-edema, and dizziness; a cough can also occur with ACEis [95]. Use of ACEis or ARBs in patients with low renal function (CKD4–5), or combination of a mineralocorticoid antagonist with ACEis or ARBs therapy, can often increase plasma potassium or result in AKI. When starting an ACEis, or increasing the dose, it is advised to check renal function and electrolytes 1–2 weeks later, with dose adjustment or medication cessation being considered if the GFR decreases by >25%, creatinine increases by >30%, or serum potassium is >5 mmol/L [95]. Patients with conditions such as CKD stage 4–5 or heart failure are at higher risk of hyperkalemia [96]. Clinicians should conduct a careful risk–benefit assessment for those patients whom double RAAS blockade is to be considered [97].

### 3.6. Statins

An inverse relationship exists between GFR and cardiovascular disease, with cardiovascular disease being the predominant cause of increased mortality in patients with CKD [6]. Renal dysfunction alters the composition of lipids to a more atherogenic profile; hypertriglyceridemia, reduced HDL cholesterol, and variable LDL and total cholesterol are seen [98]. Dyslipidemia per se is also a recognized risk for CKD and its progression [99]. Association of British Clinical Diabetologist and UK Renal Association guidelines recommend annual lipid profiles for patients with DKD [100].

Statins are 3-hydroxy-3-methylglutaryl-coenzyme A (HMG-CoA) reductase inhibitors that act to disrupt the liver’s production of cholesterol. NICE guidelines state that adults with CKD should be offered a statin to reduce their risk of cardiovascular events [8]. A renoprotective effect of statins, independent from cholesterol lowering, has also been postulated and a reduction in microalbuminuria has been observed [101,102,103,104,105]. Atorvastatin has been seen to reduce cardiovascular disease by 42% in those with GFR 30–60 mL/min/1.73 m^2^ [106]. The Association of British Clinical Diabetologist and UK Renal Association suggest the use of a statin in patients with DKD and that treatment targets should be a total cholesterol of ≤4 mmol/L, non-HDL cholesterol ≤2.5 mmol/L, and LDL cholesterol to ≤2 mmol/L [100].

#### Side-Effects

Listed side-effects of statins include myalgia, gastrointestinal symptoms, hyperglycemia, nasopharyngitis, headache, and hepatotoxicity [107]. NICE guidelines advise measuring liver function tests at 3 and 12 months after initiation of treatment. Many statins are metabolized renally and so their doses should be reduced at lower creatinine clearance levels. Statin use can cause myopathy and rhabdomyolysis that can lead to their discontinuation; however, the rates of these adverse effects are low (1.6 cases per 100,000 person years and 5 cases per 100,000 person years, respectively) [108]. If symptoms of myalgia or cramps occur then creatinine kinase levels should be measured, and if they are >5 times the upper limit of normal, then a statin should be held [107].

## 4. Recent Developments and Future Directions

**Figure 2:**
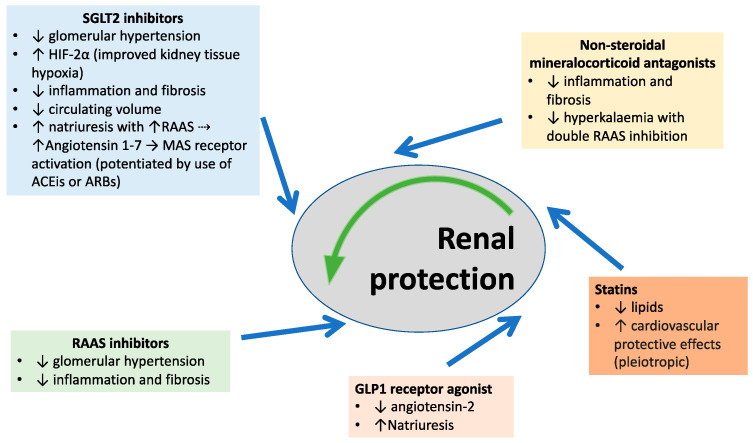
Proposed renoprotective mechanisms of action of SGLT2 inhibitors, GLP1 receptor agonists, non-steroidal MRAs, statins, and inhibitors of the RAAS (see text for detailed explanation).

Recent discoveries of new therapeutics such as SGLT2 inhibitors, GLP1 receptor agonists, and non-steroidal MRAs have taken an important role in the treatment of cardiorenal disease in the diabetic and non-diabetic population. Like in the 1990s with the introduction of RAAS inhibitors, today, we have been learning from novel molecules with cardiorenal-protective therapeutic properties that can be utilized to reduce cardiovascular morbidity and mortality (Figure 2).

The SGLT2 inhibitors, initially developed as hypoglycemic agents, have demonstrated an important cardiorenal protective effect in patients with and without diabetes [51,52,53,54,55,56,57]. The mechanism behind the hypoglycemic effect of SGLT2 inhibitors is well understood [109]. Conversely, we are still not fully aware of the processes behind their cardiorenal-protective role. The SGLT2 inhibitor-mediated cardiorenal-protective role starts within a few months from their initiation [110]. The observed rapid and beneficial cardiorenal protective effects mediated by SGLT2 inhibitors is also seen in patients without diabetes, suggesting a mechanism which is unlikely to be mediated by an amelioration in glycemic control [57,60].

Many hypotheses have been postulated. The renoprotective effect seems to be related to changes in glomerular hemodynamics. We are aware of the importance of glomerular hemodynamic perturbation in DKD [22], and how inhibition of glucose and sodium reabsorption in the proximal tubule of the nephron could lead, by tubulo-glomerular feedback, to an increase in sodium at the macula densa and secondary afferent glomerular arteriolae vasoconstriction with protection of glomerular hemodynamics [23]. This is supported by an evident fall in GFR when patients are started on a SGLT2 inhibitor [111], a phenomenon seen in most patients which represents a functional hemodynamic change and can quickly be reversed by stopping the medication.

This theory has been disputed, and studies have instead proposed that the reduction in renal vascular resistance conferred by SGLT2 inhibitors is due to post-glomerular vasodilation rather than afferent arteriolae vasoconstriction [112].

Other important mechanisms that have been postulated are the known effects of these drugs to promote erythrocytosis. An analysis from the EMPA-REG OUTCOME trial [51] suggests that an increase in hematocrit could account for more than 50% reduction in mortality observed in the study. The increase in hematocrit is seen in both the diabetic and non-diabetic population treated with SGLT2 inhibitors and has led investigators to hypothesize that this class of drugs could act via an increase in erythropoietin secretion by the kidney [113,114].

There are suggestions that SGLT2 inhibitors could act on hypoxia-inducible factors (HIF)-1α and HIF-2α, which in turn could act as mediators for the renoprotective effect [115]. Diabetic chronic kidney disease is characterized by tissue hypoxia [116], oxidative [117] and endoplasmic reticulum stress [118], and an altered and inadequate autophagic response to cellular stress [119] that lead to activation of HIF-1α and inhibition of HIF-2α.

The altered balance of HIF-1α/HIF-2α favors inflammatory and fibrotic processes as seen in both the glomerular and tubular compartment in the diabetic kidney [120]. SGLT2 inhibitors reduce oxygen consumption in the proximal tubule by inhibiting the activity of the energy dependent glucose transporter SGLT2. This results in reduced cellular stress, and enhance nutrient deprivation signaling, which contributes to inhibition of HIF-1α and stimulation of HIF-2α resulting in an increase in erythropoiesis (better tissue oxygenation) [115]. Importantly, the shift in HIF-1α/HIF-2α balance, in favor of HIF-2α, also results in reduction of renal inflammation and fibrosis. The evidence that the known hypoxia mimetic cobalt chloride mirrors the SGLT2 inhibitors effects in the kidney [115] supports this hypothetical mechanism of action for the renoprotective properties of SGLT2 inhibitors.

SGLT2 inhibitors promote natriuresis and volume depletion and activate the RAAS system [121] as seen in patients with familial glycosuria (a genetic condition characterized by mutations of the SGLT2 gene) [122]. Another proposed renoprotective effect of SGLT2 inhibitors is that, in patients treated with ACEis, angiotensin converting enzyme-2 activity predominates leading to formation of angiotensin 1–7 which in turns activate the Mas receptor promoting beneficial anti-inflammatory and anti-fibrotic effects [123].

A lot of discussion has been conducted on the observed increased ketogenesis seen with the use of SGLT2 inhibitors in clinical trials in patients with T2DM. Ketones are an efficient substrate that could benefit tissue homeostasis by relieving hypoxic stress, improving renal function, and preventing progression to CKD [124]. This theory has been challenged as ketone bodies circulating levels are increased in patients with diabetes and in diabetes the kidney is per se a ketogenic organ [125].

The renoprotective effect of GLP1 agonists is not yet fully understood. Like for SGLT2 inhibitors, data from clinical trials conducted in patients with T2DM have shown that treatment with GLP1 receptor agonists reduced the risk of cardiovascular events and slowed the development of albuminuria in diabetic patients, demonstrating a direct cardio- and renoprotective action independent of their effect on glycemic control [1,126]. GLP-1 receptor agonists seem to counteract the action of the RAAS. Infusion of GLP1 receptor agonists, in patients with diabetes, results in a reduction in plasma angiotensin-2 which has been postulated to contribute to renal protection [127,128].

Both GLP1 receptor agonists and SGLT2 inhibitors appear to have some effect on RAAS which could potentially explain their cardiorenal-protective effect. The underlying mechanisms are yet to be fully explained, and further work should focus on the interaction of these drugs on RAAS [129].

**Figure 3:**
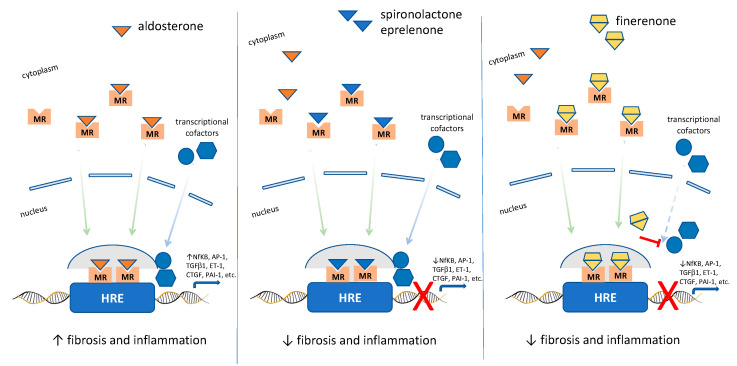
Steroidal and nonsteroidal MRAs mechanisms of action. Aldosterone binds to mineralocorticoid receptors (MR) and by translocating into the nucleus and binding to specific nuclear hormone response elements (HRE), recruits transcriptional cofactors and then initiates the transcription of target genes (e.g., NfKB, nuclear factor kappa-light-chain-enhancer of activated B cell; AP-1, activator protein-1; TGFβ1, transforming growth factor β1; ET-1, endothelin-1; CTGF, connective tissue growth factor; PAI-1, plasminogen activator inhibitor 1). Increased activation of the MR promotes proinflammatory and profibrotic processes that drive renal disease. Both steroidal and non-steroidal MRAs bind to MR inhibiting aldosterone from binding to MRs. This prevents the downstream activation of proinflammatory and profibrotic mechanisms. Steroidal MRAs, by interacting with cofactors that affect gene transcription, function as partial MR agonists. Conversely, non-steroidal MRA (e.g., finerenone) anti-inflammatory and anti-fibrotic effects are more pronounced than those of steroidal MRAs (see text for detailed explanation).

In the last few years, non-steroidal MRAs (finerenone) have been developed [130]. Steroid hormones bind their receptor which in turn interacts with many other different molecules (e.g., transcriptional cofactors) that contribute to the cellular activity of the specific ligand [131]. It has been observed that more than 300 different cofactors interact with members of the nuclear hormone superfamily. These cofactors allow ligand- and cell-type/tissue-specific cellular events that lead to different physiological responses [132]. The different cellular responses then translate into diverse actions seen between the different type of steroid and non-steroidal ligands. Modulation of the ligand (e.g., non-steroidal MRAs) results in different specific cellular/tissue effects that confer its specific mode of action [85] (Figure 3).

The non-steroidal MRA finerenone’s renoprotective effect resides in the anti-inflammatory and anti-fibrotic actions of MRA driven by RAAS inhibition [130]. Both spironolactone and eplerenone have greater accumulation in the kidney when compared to finerenone, which, in turn, has an equal distribution in the heart and kidneys. Finerenone has no active metabolites and has a short half-life which may enable a more rapid reversal of hyperkalemic episodes with this drug (especially when utilised with other RAAS inhibitors) [133]. This allows the use of finerenone for cardiorenal protection in conjunction with RAAS inhibition with ACEis or ARBs as described in the FIGARO and FIDELIO trials [86,87,88].

The mechanisms behind the cardiorenal benefit of SGLT2 inhibitors, GLP1 receptor agonists, and non-steroidal MRA are not fully understood, but it appears that the modulation of RAAS activation, as seen in disease, is central to the mechanism of action of these drugs.

Better understanding of the mechanisms behind the renoprotective properties of these recent new class of drugs is crucial. Clear knowledge of the mechanisms of action could favor the discovery of novel target for treatment and the development of new molecules. For example, alternative non-steroidal MRAs are currently being studied [134]. Some are looking into aldosterone synthesis inhibition, aldosterone gene epigenetic regulation, and targeted inhibition of specific downstream effects of the mineralocorticoid receptor [135].

Studies are also looking at potential synergistic properties of non-steroidal MRAs with SGLT2 inhibitors; in the FIDELIO-DKD trial, lower rates of hyperkalemia were observed in patients receiving SGLT2 inhibitors [136]. Other studies are looking at the combination of non-steroidal MRAs and SGLT2 inhibitors on cardiorenal outcomes [133].

Better understanding of the mechanism of action of drugs could also help towards personalized patient treatments with the identification of patients that could benefit in different way from the use of new molecules.

## 5. Summary and Conclusions

The fight against diabetic kidney disease is centred on the prevention of its development and progression. In recent years, significant new tools have become available to prevent and treat DKD. Prevention requires aggressive treatment and close follow up of patients with diabetes. Early intervention will improve patients’ health outcomes, quality of life, and health- and society-related costs. Studies aimed at better understanding the mechanisms of these new molecules may open new opportunities for patients’ personalized treatments and for the development of new therapeutic targets.
